# Transcriptome analysis provides insights into the non-methylated lignin synthesis in *Paphiopedilum armeniacum* seed

**DOI:** 10.1186/s12864-020-06931-1

**Published:** 2020-07-29

**Authors:** Lin Fang, Xin Xu, Ji Li, Feng Zheng, Mingzhi Li, Jingwei Yan, Yuan Li, Xinhua Zhang, Lin Li, Guohua Ma, Aying Zhang, Fubing Lv, Kunlin Wu, Songjun Zeng

**Affiliations:** 1 Guangdong Provincial Key Laboratory of Applied Botany, South China Botanical Garden, Chinese Academy of Sciences, Guangzhou, 510650 China; 2 University of Chinese Academy of Sciences, Beijing, 100049 China; 3 Independent Researcher, Guangzhou, 510555 China; 4 College of Life Sciences, Nanjing Agricultural University, Nanjing, 210095 Jiangsu China; 5 Environmental Horticulture Research Institute, Guangdong Academy of Agricultural Sciences, Guangzhou, 510640 China; 6 Key Laboratory of South China Agricultural Plant Molecular Analysis and Gene Improvement, South China Botanical Garden, Chinese Academy of Sciences, Guangzhou, 510650 China

**Keywords:** *Paphiopedilum armeniacum*, Lignin, Transcriptome analysis, Germination

## Abstract

**Backgrounds:**

*Paphiopedilum* is an important genus of the orchid family Orchidaceae and has high horticultural value. The wild populations are under threat of extinction because of overcollection and habitat destruction*.* Mature seeds of most *Paphiopedilum* species are difficult to germinate, which severely restricts their germplasm conservation and commercial production*.* The factors inhibiting germination are largely unknown.

**Results:**

In this study, large amounts of non-methylated lignin accumulated during seed maturation of *Paphiopedilum armeniacum* (*P. armeniacum*), which negatively correlates with the germination rate. The transcriptome profiles of *P. armeniacum* seed at different development stages were compared to explore the molecular clues for non-methylated lignin synthesis. Kyoto Encyclopedia of Genes and Genomes (KEGG) enrichment analysis showed that a large number of genes associated with phenylpropanoid biosynthesis and phenylalanine metabolism during seed maturation were differentially expressed. Several key genes in the lignin biosynthetic pathway displayed different expression patterns during the lignification process. *PAL*, *4CL*, *HCT,* and *CSE* upregulation was associated with C and H lignin accumulation. The expression of *CCoAOMT*, *F5H,* and *COMT* were maintained at a low level or down-regulated to inhibit the conversion to the typical G and S lignin. Quantitative real-time RT-PCR analysis confirmed the altered expression levels of these genes in seeds and vegetative tissues.

**Conclusions:**

This work demonstrated the plasticity of natural lignin polymer assembly in seed and provided a better understanding of the molecular mechanism of seed-specific lignification process.

## Background

*Paphiopedilum* Pftzer (Orchidacease) is commonly known as Lady’s slipper orchid because of its slipper-shaped pouch. Members of this genus have high ornamental value because the flowers are uniquely shaped and come in a wide variety of colors and sizes. Despite their horticultural value, wild populations of *Paphiopedilum* are under threats of extinction due to overcollection and habitat destruction. As a result, all species are listed in the Convention on International Trade in Endangered Species of Wild Fauna and Flora (CITES) Appendix I which also prohibit their trade [1].

Many orchid seeds including *Paphiopedilum* are tiny and contain no endosperm. In contrast, most angiosperm seeds have well-developed embryos. *Paphiopedilum* seeds are generally unable to germinate on their own. They form a mycorrhizal relationship that help with the nourishment of emerging seedling [2, 3]. Even with the additional nutrients, mature seeds of most *Paphiopedilum* species still have difficulty germinating, which severely restricts their conservation and large-scale production. However, the factors inhibiting germination are still unknown. *Paphiopedilum* seeds develop heavily lignified secondary cell walls to provide mechanical support and prevent permeability to water and nutrients [4, 5]. This feature enhances the survival of orchid seeds in harsh conditions. However, several studies imply that the accumulation of lignin contributes to the inhibition of germination during aseptic germination [6, 7]. Immature orchid seeds with low degree of lignification were shown to exhibit higher germination rates than mature seed with higher lignification [2, 8]. Mature seeds subjected to lignin degradation resulted in enhanced germination rates [2, 6, 9]. Based on these findings, it is possible that the high amounts of lignin act as germination inhibitors.

Lignin is a phenolic polymer formed by oxidative polymerization of the primary hydroxycinamyl alcohols (monolignols), ρ-coumaryl, caffeyl, coniferyl, sinapyl, and 5-hydroxylcinnamyl alcohols that give rise to ρ-hydroxylphenyl (H), catechyl (C), guaiacyl (G), syringyl (S), and 5-hydroxyguaiacyl (5H/5-OH-G) lignin. The five monolignols differ by their degree and position of methoxylation. The H and C units are non-methoxylated, whereas the G and 5H units have a single methylation on the 3-hydroxyl group and the S subunit is methylated on both the 3- and 5-hydroxyl moieties [10]. Lignin composition varies among plant species and tissue types. Lignins found in the vascular tissues are mainly composed of G and S units. However, seed lignins have a significant variation in monolignol compositions. Seed of certain species belonging to *Orchidaceae*, *Cactaceae*, *Cleomaceae*, and *Euphorbiaceae* contain lignin with the typical H, G, and S units, as well as C unit [11–13]. The significance of these variations remain unknown. The non-methylated monolignols are incorporated into lignin polymers via benzodioxane bonds, forming a linear structure without side chains [14, 15]. The linear lignin has less cross-linking with other cell wall components and is capable of enhancing the hydrophobicity and stability of the plant tissue [16]. The lignin biosynthesis pathway is well established, involving O-methyltransferases (CCoAOMT and COMT) to generate monolignols differing in their degree of methylation [10]. Disruption or downregulation of these genes can lead to changes in lignin composition, resulting in changes to cell wall integrity and mechanical properties of the tissue [17, 18].

The seeds of *Paphiopedilum armeniacum* (*P. armeniacum*) contain non-methoxylated H and C lignin. The deposition of non-methylated lignin begins in the early stages of seed development of *P. armeniacum* and eventually makes up approximately 39% of the total seed dry mass. Despite its unique structure and potentially important function, the biosynthesis of lignins in plant seeds has received little attention relative to that of vascular tissues. In this study, a detailed gene expression profiling was performed at five key developmental stages of *P. armeniacum* seeds to help elucidate the seed-specific regulation of lignin biosynthesis.

## Results

### Embryo developmental features and lignin accumulation during seed development

Zygotic embryos of *P. armeniacum* began to develop at 45 days after pollination (DAP) as previously described [3]. At 66 DAP, the T-shaped pre-embryo with four cells was formed (Table 1). At 87 DAP, the globular embryo was formed. Around 108 DAP, there is a rapid lignification of the seed coat, the inner testa disappears, and lipid globule and starch accumulate. At 122 DAP, the embryo is fully mature with a compact testa and no further observed morphological changes. Five developmental stages (66, 87, 108, 122, and 150 DAP) of *P. armeniacum* embryos were selected for further lignin structure characterization and transcriptome profiles on the basis of lignin accumulation pattern and embryo developmental characteristics.

**Table 1:** Characteristics of the developing seeds of *P.armeniacum* after pollination

DAP	Developmental status	Seed color	Germination percentage (%)	Lignin content (%)	ABA content ng/g FW	Water content (%)
66	T-shaped pre-embryo with four cells	Yellowish white	5.0 ± 0.2	6.0 ± 0.3	5.6 ± 2.7	87.0 ± 4.3
87	The globular embryo forms, and the outside-layer cells of the outer integument begin to dehydrate and the suspensor starts to degenerate	Yellowish white	44.0 ± 5.5	5.0 ± 0.9	6.5 ± 2.1	79.8 ± 2.1
108	The inner testa disappears, and starch and lipid globules accumulate rapidly	A mixture of yellow and light brown	66.0 ± 5.2	13.0 ± 3.5	9.0 ± 2.2	72.2 ± 1.1
122	The embryo is fully mature with a compact testa and no further morphological changes	Brown dark	37.0 ± 7.2	22.0 ± 4.1	14.4 ± 3.1	68.6 ± 2.6
150	Dry and mature seeds	Dark	10.0 ± 1.7	39.0 ± 5.45	1.4 ± 0.2	51.5 ± 0.6
180	Dry and mature seeds	Dark	5.0 ± 0.75	37.0 ± 3.56	2.3 ± 1.2	49.2 ± 0.7

**Fig. 1:**
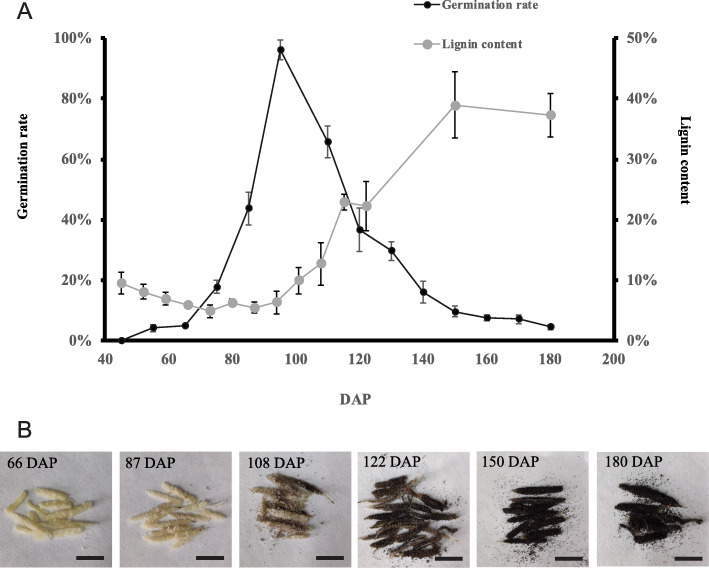
Dynamic changes in seed morphology, lignin accumulation, and germination rate during *P. armeniacum* seed development. **a** Lignin content and germination rate during *P. armeniacum* seed development. Days after pollination (DAP). The lignin content was estimated using the acetyl bromide method. **b** Phenotypes of *P. armeniacum* seeds. Scale bar = 1 cm

The germination was scored together with the lignin content during the seed development (Fig. 1a). During the early stages of seed development (45–95 DAP), the germination rate gradually increased as the embryo formation. At 95 DAP, the globular embryo has been fully developed, and the germination rate reached 96.2%. During this stage, lignin was maintained at a relatively low level (5–9% of the dry mass). After that, even though the starch and lipid globules continue to accumulate as described in a previous study [3], the germination rate dropped sharply from 96 to 5%. Meanwhile, the amounts of lignin started to increase rapidly, with the seed coat changing from white to brown (Fig. 1b). Lignin accumulation reached a plateau (~ 39% of the dry mass) at 150 DAP, with the seed coats becoming dark and hard.

### Identification of non-methylated lignin in seeds

To analyze the monolignol composition from various tissue types, the samples were subjected to pyrolysis-gas chromatography/ mass spectrometry (Py-GC/MS). The identities and relative molar abundances of the released compounds are listed in Table 2. The results showed that the lignin in the seeds contain high levels of C units and H units without any traces of G and S units. In contrast, lignins present in other tissues (stems, leaves, and pods residues after seed isolation) were composed mainly of G and S units with essentially no C units.

**Table 2:** Relative molar abundances (%) of the compounds released after pyro-GC/MS of seeds, stems, leaves and pods of *P. armeniacum*. Values in brackets are the standard deviations from three biological replicates

Compound name	Origin	Formula	Molecular Mass	Main Mass fragments	Seeds (%)	Stems (%)	Leaves (%)	Pods (%)
Phenol	H	C_6_H_6_O	94	65,66,94	16.2 (0.1)	–	23.0 (6.9)	5.7 (1.2)
2-Methylphenol	H	C_7_H_8_O	108	77,107,108	18.7 (0.4)	–	–	–
3-Methylphenol	H	C_7_H_8_O	108	77,107,108	18.3 (0.4)	–	–	–
2,5-Dimethylphenol	H	C_8_H_10_O	122	77,107,122	7.2 (1.3)	–	–	–
Catechol	C	C_6_H_6_O_2_	110	53,64,110	19.6 (2.7)	–	–	–
4-Methylcatechol	C	C_7_H_8_O_2_	124	51,97, 125	13.4 (0.7)	–	–	–
4-Ethylcatechol	C	C_8_H_10_O_2_	138	51,78,124	6.5 (0.1)	–	–	–
2-Methoxylphenol	G	C_7_H_8_O_2_	124	81,109,124	–	14.9 (0.8)	16.1 (2.1)	19.0 (2.1)
2-Methoxy-5-methylphenol	G	C_8_H_10_O_2_	138	95,123,138	–	5.6 (0.2)	–	13.4 (2.7)
4-Ethenyl-2-methoxyphenol	G	C_9_H_10_O_2_	150	107,135,150	–	31.6 (4.6)	23.1 (3.2)	31.7 (3.2)
4-Hydroxy-3-methoxybenzaldehyde	G	C_8_H_8_O_3_	152	109,151,152	–	4.5 (0.1)	–	5.5 (0.3)
2-Methoxy-4-propenylphenol	G	C_10_H_12_O_2_	164	131,149,164	–	7.9 (0.1)	–	11.2 (0.6)
4-Hydroxy-3-methoxyphenyl acetone	G	C_10_H_12_O_3_	180	122,137,180	–	4.1 (0.3)	18.1 (0.7)	–
2,6-Dimethoxyphenol	S	C_8_H_10_O_3_	154	111,139,154	–	–	19.8 (4.7)	13.6 (2.6)
4-Hydroxy-3,5-dimethoxystyrene	S	C_10_H_12_O_3_	180	137,165,180	–	31.6 (2.7)	–	–
% H units					60.5 (2.0)	–	23.0 (4.3)	5.7 (0.9)
% C units					39.5 (2.0)	–	–	–
% G units					–	68.4 (6.9)	57.2 (3.1)	80.7 (2.5)
% S units					–	31.6 (2.1)	19.8 (2.9)	13.6 (1.9)

**Fig. 2:**
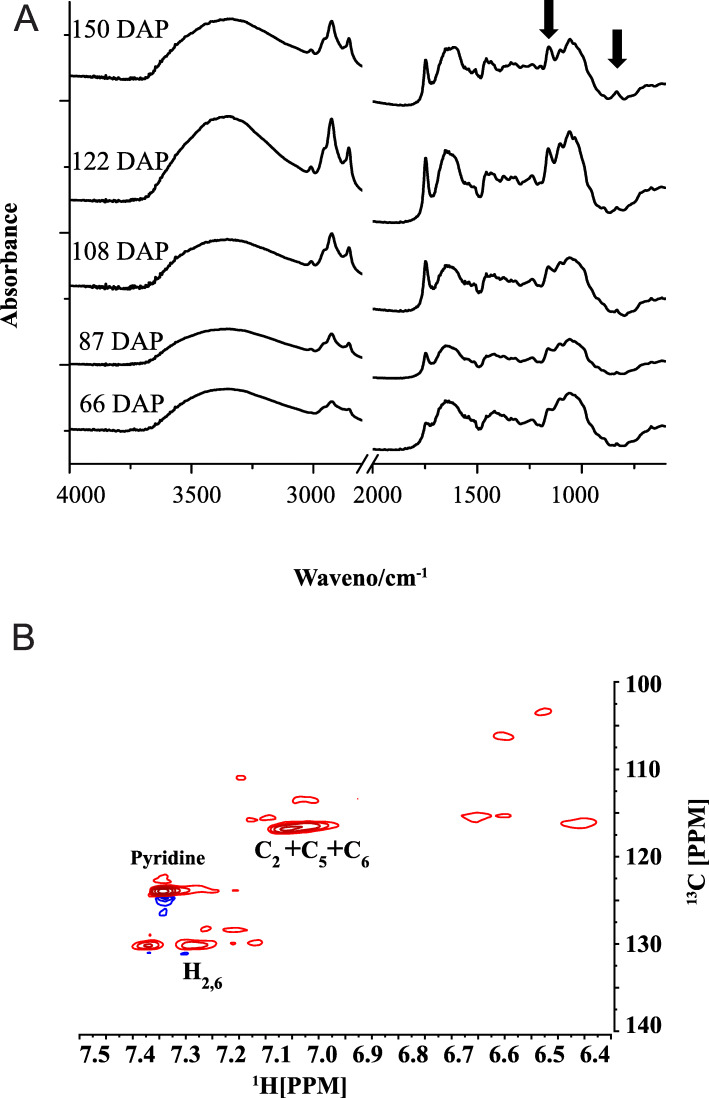
Lignin structure characterization. **a** FTIR spectra of *P. armeniacum* seeds at five developmental stages. Positions of C lignin bands are marked by arrows. **b** Partial short-range ^13^C–^1^H (HSQC) spectra (aromatic region) of 122 DAP mature seeds

The composition of monolignol structure in seeds was further characterized for the assignments for C lignin and G/S lignin in Fourier transform infrared spectroscopy (FTIR) and Nuclear Magnetic Resonance (NMR) spectra in orchid seeds as previously established [11–13]. FTIR spectra from the seeds at five developmental stages indicated C lignin deposited in the early stage with distinct C lignin bands at 1154 cm^− 1^ and 823 cm^− 1^ (Fig. 2a). The rapid development of the two bands was observed at 108 DAP. In agreement with Py-GC/MS analysis, no typical G/S lignin was detected in *P. armeniacum* seeds during the five developmental stages. Analysis of the aromatic region of the 2D NMR spectra of mature seeds confirmed the presence of H and C lignin (Fig. 2b). The results indicated that the monolignol composition varied among tissue types in *P. armeniacum*, and that the non-methylated C lignin and H lignin specifically deposited in the seed.

### RNAseq analysis, de novo assembly, and functional annotation

RNA-seq analysis of *P. armeniacum* seeds was performed at five developmental stages (66, 87, 108, 122, and 150 DAP) to characterize the transcriptome dynamics of *P. armeniacum* seed development and to identify genes involved in the seed-specific lignification. Three biological replicate sequencing libraries were prepared from each stage. A total of 104.12 Gb clean data were generated from each library after filtering out low-quality data. The transcriptome details for each sample are shown in Table S1. Q30 of the raw data ranged from 92.10 to 94.11% indicating high-quality reads worthy of further analysis. Since no reference genome is available for *Paphiopedilum*, a de novo assembly of all 347,075,856 reads into 433,854 transcripts with an N50 length of 1180 bp and 183,737 unigenes with an average length of 860 bp. The transcripts and unigenes length distribution are shown in Table S2. The biological reproducibility was assessed using the Pearson’s correlation coefficient, which showed that the correlation between samples among the same biological replicates was high (Fig. S1A). Principle component analysis (PCA) of all samples during seed development was also performed (Fig. S2B). Consistent with their distinct developmental stages, samples from different biological replicates were clustered separately.

**Fig. 3:**
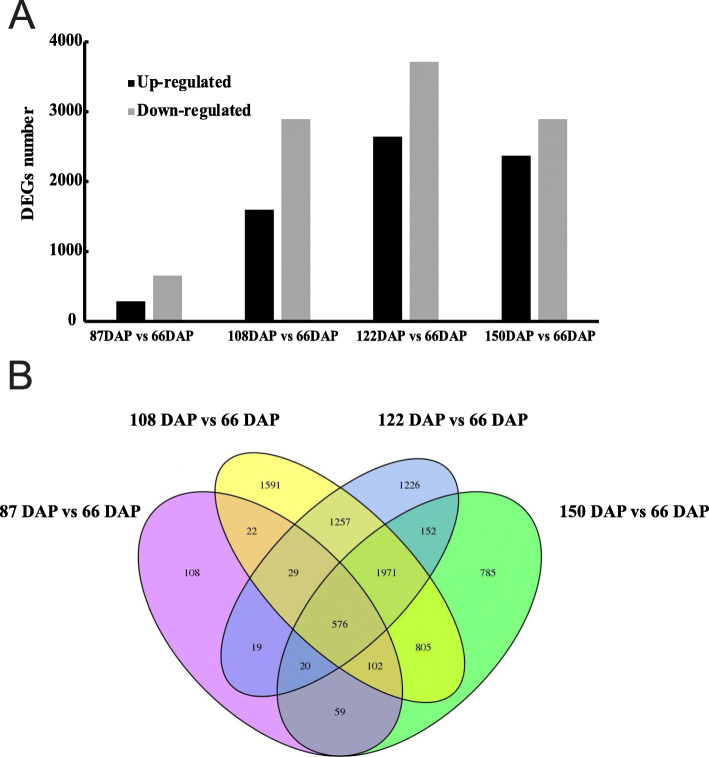
Statistical analysis of differentially expressed unigenes (DEGs) during *P. armeniacum* seed development. **a** Up/down-regulated unigenes in all development stages. The total DEG number peaked between 122 DAP and 66 DAP. **b** Venn diagram of all DEGs

For annotation, 183,737 unigenes were subjected to BLASTX searches against the sequences in the NCBI non-redundant protein sequences (NR), Swissprot, Gene Ontology (GO), the Clusters of Orthologous Groups (COG), and Kyoto Encyclopedia of Genes and Genomes (KEGG) databases. As a result, a total of 92,235 unigenes (50.20% of all unigenes) could be assigned at least one putative function from one of these databases (Table S3). A total of 89,285 unigenes were annotated from the NR database. Further analysis of the matched sequences showed that the *P. armeniacum* transcript was highly similar to *Dendrobium catenatum* (29.86%), *Apostasia shenzhenica* (8.38%), and *Phalaenopsis equestris* (5.45%) as shown in Fig. S2A. For GO annotation, a total of 29,301 unigenes from *P. armeniacum* seeds were annotated into three GO pathways, as shown in Fig. 3b. The functions of unigenes in biological process classifications contained cellular process, metabolic process, and biological regulation. Cell part, cell, and organelle were the most abundant functions in terms of cellular component classifications. The most abundant biological process functions are metabolic process and cellular process. In the molecular function classification, binding and catalytic activity were more abundant. The main GO entries were consistent with the fact that the cells divided frequently during seed development. Other entries such as catalytic, metabolic and binding activities were relatively high.

### Differential gene expression and KEGG enrichment analysis

With the restrictive conditions of False Discovery Rate (FDR) < 0.05 and log2 ratio ≥ 1.0, unigenes that were differentially expressed in the seeds at the five developmental stages were identified. In total 8722 differentially expressed genes (DEGs) were identified among all the libraries. The number of DEGs increased with seed development and peaked among 122 DAP and 66 DAP seeds (Fig. 3a). A total of 576 common DEGs were identified in 87 DAP, 108 DAP, 122 DAP, and 150 DAP among all developmental stages, implying that these DEGs might be associated with seed development (Fig. 3b).

**Fig. 4:**
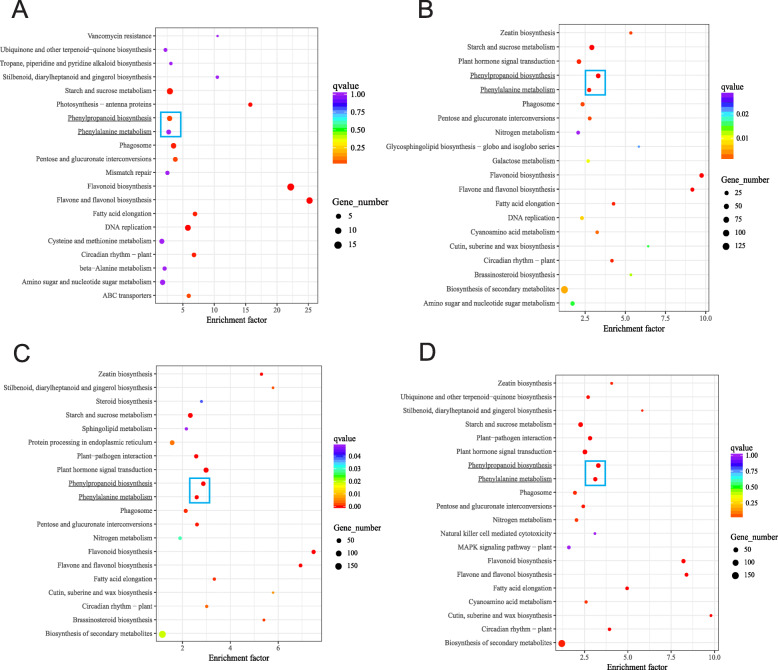
KEGG enrichment analysis with the DEGs. **a** 87 DAP vs 66 DAP, **b** 108 DAP vs 66 DAP, **c** 122 DAP vs 66 DAP, (D) 150 DAP vs 66 DAP. Flavone and flavonol biosynthesis (ko00944) and flavonoid biosynthesis (ko00941) are highly enriched throughout the five key development stages. Phenylpropanoid biosynthesis (ko00940) and phenylalanine metabolism (ko00360) were enriched during the seed lignification

KEGG enrichment analysis with the DEGs in 87 DAP vs 66 DAP, 108 DAP vs 66 DAP, 122 DAP vs 66 DAP, and 150 vs 66 DAP (Fig. 4 and Table S4) were performed to provide more insight into the DEGs regulating lignin deposition at the later stages of seed development. The top-enriched KEGG pathways of these DEGs were secondary metabolite biosynthesis pathways, including flavonoid, phenylpropanoid and flavone, and flavonol biosynthesis pathways, etc. Among them, flavone and flavonol biosynthesis (ko00944) and flavonoid biosynthesis (ko00941) are highly enriched throughout the five key developmental stages, suggesting these pathways are potentially important for seed development such as with pigment accumulation, but not specifically related to lignin deposition. Our premise is that genes involved in non-methylated lignin accumulation would be induced primarily during the lignin rapid accumulation stage (from 101 DAP to 150 DAP). The analysis showed that phenylpropanoid biosynthesis (ko00940) and phenylalanine metabolism (ko00360) are indeed highly enriched in 108 DAP vs 66DAP, 122DAP vs 66DAP, and 150 vs 66DAP. Other highly enriched pathways were DNA replication (ko03030) in 87DAP vs 66DAP, starch and sucrose metabolism (ko00500) in 87DAP vs 66DAP and 108DAP vs 66DAP, and plant hormone signal transduction (ko04075) in 150DAP vs 66DAP.

### Identification of genes potentially involved in non-methylated lignin biosynthesis

**Fig. 5:**
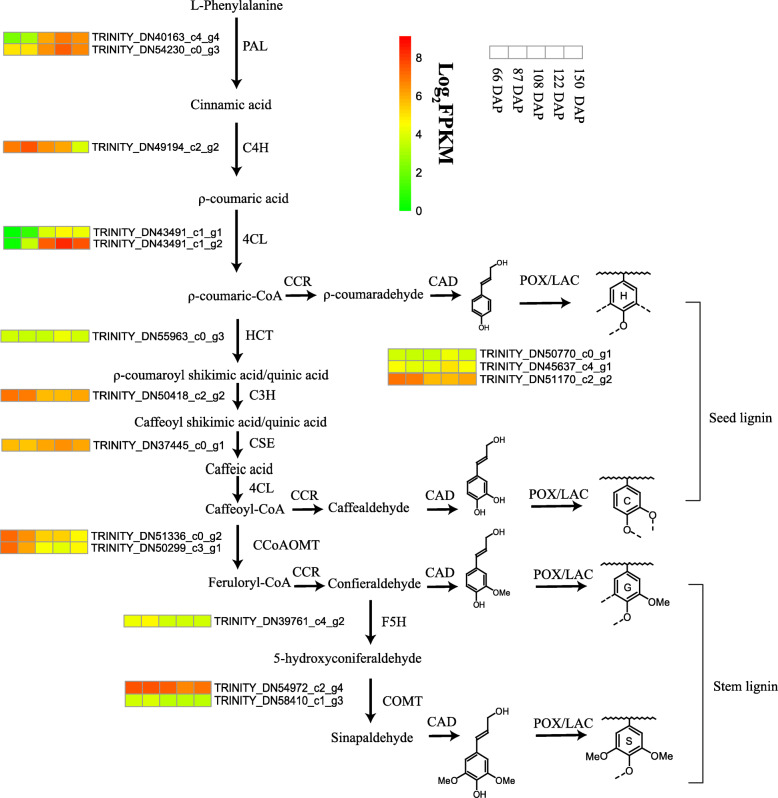
Lignin biosynthesis pathway and monolignol biosynthesis related gene expression in *P. armeniacum*. PAL, phenylalanine ammonia lyase; C4H, cinnamate 4-hydroxylase; 4CL, 4-coumarate:CoA ligase; CCR, cinnamoyl-CoA reductase; CAD, cinnamyl alcohol dehydrogenase; C3H, 4-coumarate 3-hydroxylase; HCT, hydroxycinnamoyl-CoA:shikimate/quinate hydroxycinnamoyl transferase; CSE, caffeoyl shikimate esterase; CCoAOMT, caffeoyl-CoA 3-O-methyltransferase; COMT, caffeic acid O-methyltransferase; F5H, ferulate-5-hydroxylase; CAD, cinnamyl alcohol dehydrogenase; POX/LAC, peroxidases/laccase. Gene expression was scaled using the Z-score of FPKM (mean value of three biological replicates) in the heatmap. For each heatmap, the key is located at right side with FPKM values increasing from green to red

**Fig. 6:**
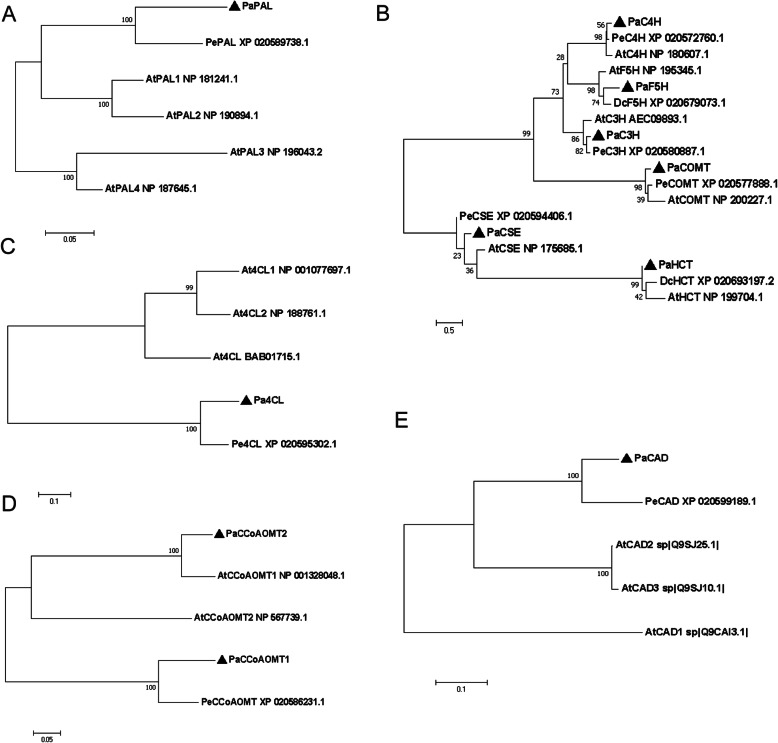
Phylogenetic analysis of monolignol pathway gene candidates among different plant species. The phylogenetic trees were constructed using maximum likelihood method with MEGA 7 software (Molecular Evolutionary Genetics Analysis version 7.0) with 1000 bootstrap replicates. Analysis of C4H, C3H, F5H, COMT, HCT, and CSE was processed together, because at least four taxa are needed for bootstrapping. **a** PAL, phenylalanine ammonia lyase; **b** C4H, cinnamate 4-hydroxylase; C3H, 4-coumarate 3-hydroxylase; F5H, ferulate-5-hydroxylase; COMT, caffeic acid O-methyltransferase; CSE, caffeoyl shikimate esterase; HCT, hydroxycinnamoyl-CoA:shikimate/quinate hydroxycinnamoyl transferase; **c** 4CL, 4-coumarate:CoA ligase; CCR, cinnamoyl-CoA reductase; CAD, cinnamyl alcohol dehydrogenase; **d** CCoAOMT, caffeoyl-CoA 3-O-methyltransferase; **e** CAD, cinnamyl alcohol dehydrogenase

The monolignol biosynthesis pathway is well established, and a series of enzymatic reactions catalyzed by specific enzymes have been identified, including phenylalanine ammonia lyase (PAL), cinnamic acid 4-hydroxylase (C4H), 4-coumarate: CoA ligase (4CL), cinnamoyl-CoA reductase (CCR), cinnamyl alcohol dehydrogenase (CAD), hydroxycinnamoyl CoA: shikimate hydroxycinnamoyl transferase (HCT), 4-coumarate 3-hydroxylase (C3H), caffeoyl shikimate esterase (CSE), ferulic acid 5-hydroxylase (F5H), caffeic acid O-methyltransferase (COMT), and caffeoyl-CoA 3-O-methyltransferase (CCoAOMT) [10, 15] (Fig. 5). The annotated lignin pathway gene in the KEGG database identified 20 *Paphiopedilum* genes that are homologous to genes potentially involved in monolignol synthesis (Table S5). Phylogenetic trees for the individual protein families of Arabidopsis, Dendrobium, and Phalaenopsis were constructed using the CDS sequenced listed in Table S10. The results showed that the identified genes in lignin biosynthetic pathways had a close genetic relationship with those reported in other plant species (Fig. 6).

**Fig. 7:**
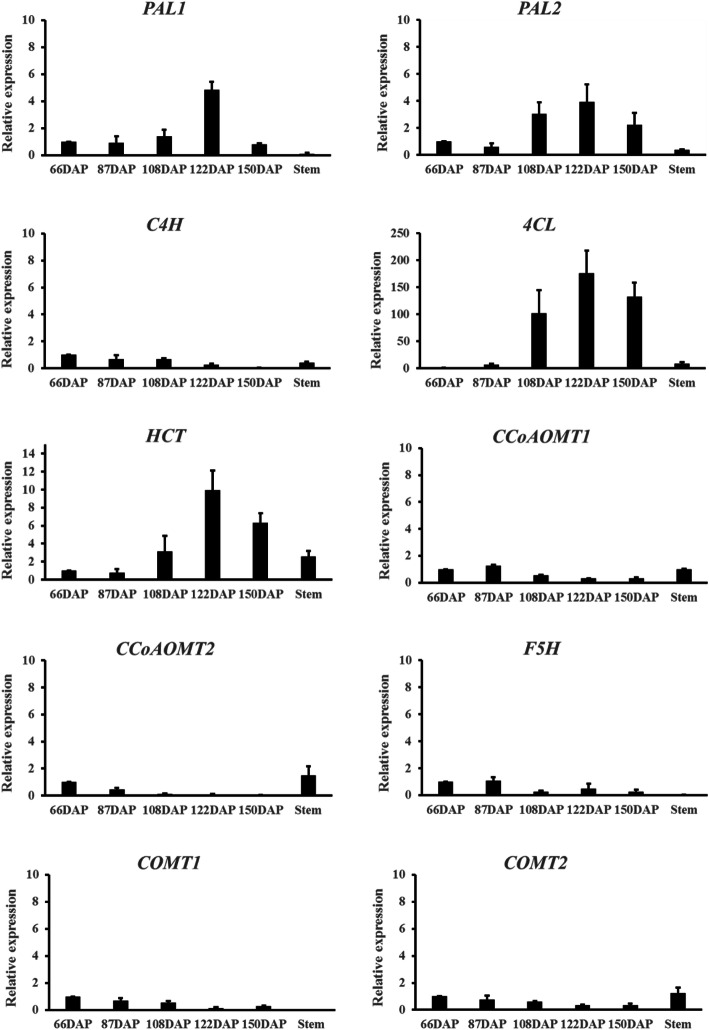
Validation of lignin-related gene expression in five developmental stages of seed and stems. Expression levels were normalized to expression levels of actin

Most lignin-related genes displayed a very similar transcript level between 66 DAP and 87 DAP, prior to the rapid accumulation of lignin (Figs. 6 and 7). Several monolignol synthesis related genes *PAL*, *4CL*, *HCT,* and *CSE* demonstrated increased expression levels from 87 DAP to 150 DAP. Despite the high expression levels of these genes, most *CCoAOMT*, *COMT,* and *F5H* were absent or down-regulated from developing seeds, and a few displayed a significant decrease in the expression level. *CCoAOMT* expression level is relatively high at 66 DAP, but the low expression level of *F5H* made the conversion of caffeoyl moieties to feruloyl moieties inefficient, which resulted in no G and S lignin production at the early stage of the seed development. Later, the decrease in the expression level of *CCoAOMT* and upregulation of upstream genes in monolignol pathway caused the accumulation of H and C lignins instead of G and C lignins.

### qRT-PCR validation of candidate genes involved in lignin biosynthesis

Contigs corresponding to *PAL*, *4CL*, *HCT*, *C4H*, *CCoAOMT*, *F5H,* and *COMT* in five development stages of seed development were selected for qRT-PCR validation to verify the accuracy and reproducibility of the RNA-seq results. Methylated lignin-rich tissue stems were also included to verify our predicted monolignol pathway. The sequences of the primers used are given in Table S6. Reliable estimation of gene expression by qRT-PCR requires stable reference genes for data normalization. Six housekeeping genes *ACTIN1*, *ACTIN2*, *TUBLIN*, *UBIQUITIN*, *RPS3A1,* and *RPS3A2* were tested for suitability as reference genes in the five development stages of seeds, leaves, and stems. qRT-PCR was performed using the primers listed in Table S8 to evaluate the expression variation of these candidates across seeds and vegetative tissues. The average threshold (C_T_) values of the candidate genes ranged from 17.25 to 23.66 in seeds and vegetative tissues (Table S9). The coefficient of variation (CV) and ΔC_T_ are parameters used to identify suitable reference genes. The lower the values, the more stable the gene expression. *ACTIN1* and *ACTIN2* were the most stable genes among the six candidate reference genes. Therefore, *ACTIN1* was chosen as a reference gene for this study. The additional experimental details on qRT-PCR are listed in Fig. S3 to demonstrate the reliability of the assay.

Consistent with the transcriptomic data, *PAL*, *4CL,* and *HCT* showed increased expression level as the seed approached maturity (Fig. 7). *CCoAOMT* displayed relatively high transcript levels at the early stages of seed development and low transcript levels during the later stages. *F5H* and *COMT* were also down-regulated during seed development. The results confirm the reliability of the transcriptomic data. Stems displayed relatively high transcript levels of *CCoAOMT* in contrast to the mature seeds, which suggests that down-regulation of the first lignin methylation gene *CCoAOMT* is crucial for non-methylated lignin production.

## Discussion

The structure of orchid seeds is quite simple with its balloon-like seed coat, globular embryo, and lack of endosperm. In comparison, most angiosperm seeds have a well-developed embryo and endosperm. Orchid seeds, often referred to as “dust seeds,” are typically dispersed by wind [19]. Most species of orchid have difficulty germinating under natural conditions. *P. armeniacum* is one of the most difficult species to propagate, which severely restricts its large-scale production. The factors that inhibit germination are still largely unknown. Plant hormones play essential roles in seed development and germination performance. Abscisic acid (ABA) is necessary for inducing the synthesis of reserve proteins and lipids, as well as the desiccation of developing seeds [20, 21]. In *Cypripedium formosanum*, an orchid species with low germination rates, ABA was proposed to be the key germination inhibitor [22]. In *P. armeniacum* seed development, ABA content was increased to 14.4 ng/g at 122 DAP from 5.6 ng/g at 66 DAP (Table 1). It is possible that increased levels of ABA inhibit germination and promote desiccation before the seeds are fully mature. At 150 DAP, the ABA level dropped to 2.3 ng/g with continued decrease on germination rate. Exogenous applications of ABA or fluridone, an ABA inhibitor, can change the ABA levels within the seeds, but no significant differences on germination rate was observed (data not shown). Therefore, we believe that other factors may be contributors to the inhibition of germination.

Mature orchid seeds generally have a heavily lignified seed coat that provides protection to the embryo. Several studies attributed lignin accumulation to germination inhibition of mature seeds [2, 6, 9]. In this study, the lignin content was measured during the seed development process to verify whether the lignin content correlates with the germination rate. During the early stages of development, the germination rate gradually increased as the embryo became fully developed, with lignin content maintained at low levels. The germination rate abruptly dropped when the seed lignin accumulated rapidly around 95 DAP. The results showed a negative correlation with lignin amount and germination rate. One possibility is that the lignin-rich seed coat provided mechanical restraint by preventing water and nutrients uptake [4].

The structure and composition of lignin have also been shown to impact the cell wall mechanical properties [23]. In this study, lignins in the *Paphiopedilum* seeds are composed of H and C units, as clearly evident from Py-GC, two-dimensional NMR, and FTIR analysis (Table 2 and Fig. 2), while the lignins in other parts of the *Paphiopedilum* plant are composed of typical G and S units. This discovery raised an important question: what is the mechanism for regulating the seed specific deposition of non-methylated lignin?

Five key stages were selected for transcriptome analysis based on the dynamics of lignin accumulation and key anatomical features in embryo development. A total of 104.12 Gb clean data were obtained from 15 RNA-seq libraries of 66, 87, 108, 122, and 150 DAP. The assembled data had an average N50 length of 1180 bp, which is similar to that in other orchid species, such as *Dendrobium catenatum* and *Apostasia shenzhenica* [24, 25]. Phenylpropanoid biosynthesis and phenylalanine metabolism are highly enriched during the rapid lignin accumulation stages. Further DEGs analysis of phenylpropanoid biosynthesis pathway showed that major structural genes were upregulated except for *CCoAOMT*, *F5H,* and *COMT.* Both CCoAOMT and COMT catalyze the O-methylation of the C3 and C5 hydroxyl groups of lignin precursors during G and S lignin formation. Earlier studies demonstrated low O-methyltransferase (CCoAOMT and COMT) activities in the C-lignin containing seeds of *Cleome hasslerianan* (*C. hasslerianan*) [17]. CCoAOMT is the first O-methyltransferase in monolignol pathway. Caffeyl alcohol would be formed if CCoAOMT became depleted or lost its function. Down regulation of *CCoAOMT* in *Pinus radiata* introduces caffeyl alcohol into lignification, resulting in low levels of C units present in the G lignin dominant tissues [18]. In this study, the low expression level of *CCoAOMT*, *COMT,* and *F5H* suggest it is possible that the biochemical basis for the accumulation of non-methylated lignin in seeds. In stem tissues where G and S lignins are abundant, the expression levels of *CCoAOMT2* and *COMT2* are significantly higher than those in the seeds (Fig. 7). Suppression or loss of function of *CCoAOMT* and *COMT* in several angiosperm species such as Arabidopsis, alfalfa, poplar, and tobacco did not result in the incorporation of caffeyl alcohol [26–28]. In addition to the methylation genes (*CCoAOMT*, *COMT,* and *F5H*), biosynthesis of C lignin requires the expression of Cinnamyl alcohol dehydrogenase (CAD) with a preference for the caffealdehyde in the seed coat of *Cleome hassleriana* [29]. CAD catalyzes the conversion of the corresponding cinnamyl aldehydes to cinnamyl alcohols, which is the last step in the synthesis of monolignols before their polymerization on to the cell walls. However, none of the CAD genes were expressed in a manner consistent with the involvement of C-lignin formation in *P. armeniacum*.

Other factors may also determine the lignin composition, such as transcriptional regulation or availability of specific monolignol transporters. NAC and MYB families have been identified as regulators of lignin deposition [10, 30, 31]. In our RNA-seq dataset, a total of 944 unigenes that encode TFs associated with 54 families were identified from our assembled transcripts. A total of 40, 188, 274, and 247 differentially expressed TFs were found in 87 DAP vs 66 DAP, 108 DAP vs 66 DAP, 122 DAP vs 66 DAP, and 150 vs 66 DAP, respectively (Table S6). Instead of the master switches, this study is more focused on the TFs that can specifically regulate the methylation genes, since these TFs can control the accumulation of non-methylated lignin. A few MYBs were identified as potential regulators of *CCoAOMT*, such as MYB4 and MYB21 (Fig. S4). *MYB4* was down-regulated as the seed approached maturity. *MYB4* is a positive regulator of *CCoAOMT* and a negative regulator of *PAL*, *C4H*, and *4CL* in Arabidopsis and *Pinus tadeda* [32, 33]. *MYB21* is a negative regulator of *CCoAOMT*, and the expression level of other structural genes remains unchanged under the influence of MYB21 in Poplar [31]. The expression of these TFs is in agreement with the expression of major structural genes involved in monolignol pathways, implying a potential role in lignin synthesis regulation.

## Conclusion

In summary, the presence of H and C lignin in *Paphiopedilum* seeds exemplifies the flexibility of lignin monomer assembly in nature. Transcriptome profiling revealed the lignin-related candidate genes expression pattern, which clarified the seed-specific monolignol pathways. However, the seed monolignol composition is not consistent even within the same families of *Cypripedium*, *Euphorbiaceae*, and *Cleomaceae* plants, suggesting the feature is not conserved. The difference in the degree of methylation and molecular structure of C and H lignin strongly suggests a functional difference compared to typical G and S lignin. Future work should focus on linking these unique non-methylated lignin structures to their mechanistic role for seed development and germination.

## Methods

### Plant materials and growth conditions

*P. armeniacum* S. C. Chen et F. Y. Liu plants were maintained in a glass greenhouse in the South China Botanical Garden, Guangzhou, China. The plants were potted in a substrate of Zhijing stone for orchids under 800 μ mol m^− 2^ s^− 1^ natural light maintained with a sunshade net. The average temperature and relative humidity ranged from 10 to 32 °C and 70–98%, respectively. The flowers from three-year-old adult plants were labeled and artificially self-pollinated. Capsules of different developmental stages from 45 DAP to 180 DAP were collected at 7-day intervals from grown plants. Seeds were removed from capsules and immediately frozen in liquid nitrogen.

### Seed germination assay

Seed capsules were surface sterilized as previously described [34]. Seeds were sown on Hyponex N026 medium supplemented with 1.5 g/L activated charcoal, 2 g/L peptone, 15 g/L sucrose, and 5% coconut water. Germination was scored after the embryo swelled and the testa ruptured.

### ABA content

ABA content was measured as previously described [35]. About 100 mg of seeds were used for ABA quantification by high-performance liquid chromatography–mass spectrometry.

### Water content

Seeds after capsule removal were dried at 100 °C until constant dry weight was achieved (40–48 h). Water content was expressed as a percentage of fresh weight.

### Lignin content and composition

Lignin content was estimated using acetyl bromide as previously described but with slight modifications [36]. Five (5) mg of finely ground seed was thoroughly extracted with ethanol/toluene (1:1) until the extracts no longer absorbed UV light at 280 nm. The dried samples were placed into loosely capped glass tubes containing 1 mL of acetyl bromide/acetic acid (1:3) and incubated at 70 °C for 30 min. The samples were then cooled in an ice bath and mixed with 0.9 mL of 2 M NaOH, 5 mL glacial acetic acid, and 0.1 mL 7.5 M hydroxylamine hydrochloride. The final volume was adjusted to 10 mL with glacial acetic acid and the absorbance measured at 280 nm with a microplate reader (Tecan Infinity). A standard curve was generated using alkali lignin (Sigma-Aldrich, 471,003) for lignin content calculation.

The chemical composition of lignin was analyzed by Py-GC/MS using a previously described method with some modifications [37]. The pyrolysis was performed on a Pyroprobe 5000 (CDS Analytical Inc.) directly connected to a Shimadzu GCMS-QP2010A equipped with a HP-5MS column (30 m × 0.25 mm × 0.25 mm). The pyrolysis was carried out at 550 °C. The chromatograph was programmed from 50 °C to 250 °C at a rate of 15 °C/min and the final temperature was held for 10 min. Helium was used as the carrier gas at a constant flow rate of 1 mL/min. The mass spectrometer was operated in scan mode and the ion source was maintained at 300 °C. The compounds were identified by comparing their mass spectra with those of the NIST library and those previously reported [38, 39]. Peak molar areas were used to calculate the lignin degradation products, and the summed areas were normalized. The relative abundance was calculated from the summed peak areas of each unit.

### FTIR

The dried samples were embedded in KBr pellets in the concentration of about 1 mg/100 mg KBr. FTIR spectra in the range of 4000–400 cm^− 1^ were recorded using a Shimadzu IRAffinity-1S FTIR spectrophotometer. The spectra were recorded in the absorption mode at 64 scans per sample with a resolution of 4 cm ^− 1^.

### 2D ^13^C-^1^H heteronuclear single-quantum coherence (HSQC) NMR spectroscopy

Seeds from 122 DAP were extracted and ball-milled as previously described [40]. The gels were formed using DMSO-d_6_/pyridine-d_5_ (4:1) and sonicated until homogenous. The homogeneous solutions were transferred to NMR tubes. HSQC spectra were acquired at 25 °C using a Bruker Avance-500 MHz. The running conditions and assignment of the spectra were followed as previously described [13].

### RNA extraction, library construction, and RNA sequencing

The five developmental stages analyzed were 66, 87, 108, 122, and 150 DAP, with three biological replicates per developmental stage. The total RNA was extracted using Column Plant RNAout2.0 (Tiandz Inc., Beijing, China) according to the manufacturer’s protocol, which was specifically designed for materials rich in polysaccharides and polyphenolics. Extracted RNA was treated with DNase (Tiandz Inc., Beijing, China) to remove genomic DNA. The RNA quality was validated using agarose gel electrophoresis, Nanodrop One (Nanodrop Technologies Inc., DE, USA), and Agilent 2100 (Agilent Technologies Inc., CA, USA) to confirm the purity, concentration, and integrity, respectively. Library construction and sequencing were performed using Illumina HiSeq4000 platform (Illumina Inc., CA, USA) by Genepioneer Technologies Corporation (Nanjing, China).

### De novo assembly, functional annotation of unigenes, and DEGs analysis

The clean data was generated by removing adaptor sequences, ambiguous reads (‘N’ > 10%), and low-quality reads (greater than 50% of bases in a read with a quality value Q ≤ 5) using perl script. All sequence data were uploaded into the BioProject database hosted by the National Center for Biotechnology Information (NCBI) under the BioProject PRJNA550294. The transcripts were assembled using Trinity v2.4.0 program with default parameters [41], and gene expression was estimated by applying the fragments per kilobase per million mapped reads (FPKM). Functional annotations were performed using public databases, including the NR [42], Swiss-Prot [43], COG [44], and GO [45]. Differentially expressed genes (DEGs) between libraries were identified as those with the fold change (FC) of the expression level (FC ≥ 2 or FC ≤ 0.5 under *P*-value ≤0.05, FDR ≤ 0.05). GO enrichment and KEGG enrichment were performed using the obtained DEGs. To identify genes encoding for TFs, all DEGs were compared with protein sequences downloaded from the plant TF database, PlantTFDB, with the E-values threshold of 10^− 10^.

### Verification of gene expression using qRT-PCR

Samples of RNA-seq were reversed transcribed into cDNA for real-time qPCR validation using GoScript Reverse Transcription System (Promega, CA, USA). The qRT-PCR reactions were performed on ABI 7500 Real-Time PCR System (Applied Biosystems, CA, USA) using the SYBR Premix ExTaq Kit (Takara, Dalian, China). Primer sequences are listed in Table S7. The expression level was calculated as 2^-ΔΔCt^ and normalized to the Ct values of *P. armeniacum Actin* (TRINITY_DN40678_c2_g1).

### Phylogenetic analysis

The phylogenetic trees were constructed using maximum likelihood method with of MEGA 7 software (Molecular Evolutionary Genetics Analysis version 7.0) with 1000 bootstrap replicates. Analysis of C4H, C3H, F5H, COMT, HCT, and CSE were processed together because at least four taxa are needed for bootstrapping.

### Statistical analysis

Statistical analysis was performed using Student’s *t*-test as indicated in the figure legends. All experiments were performed on independently grown biological replicates. All values represent the mean ± SD.

## Supplementary information

**Additional file 1: Figure S1.** (A) Correlation indices between different samples. (B) Principal component analysis performed on the 15 samples. A: 65 DAP, B: 87 DAP, C: 108 DAP, D: 122 DAP, E:150 DAP. Figure S2. Functional annotations of the unigenes of *P. armeniacum* seed transcriptome. (A) NR annotated species distribution map similar to the *Paphiopedilum armeniacum* transcriptome. *Dendrobium catenatum* shows the highest similarity. (B) GO function annotation. The most abundant functions are binding and catalytic activity in terms of molecular function and metabolic process and cellular process in terms of biological process. **Figure S3.** Additional data for qPCR assays. (A) The quality of RNA used for qPCR assessed by agarose gel electrophoresis. 1–4: 66 DAP, 5–8:87 DAP, 9–12: 108 DAP, 13–16: 122 DAP, 17–20: 150 DAP. Lanes 1 and 2 are the 28S and 18S RNA bands. (B) Quantification of RNA used for qPCR estimated by Nanodrop One and Agilent 2100 Bioanalyzer. (C) Melt curve from qPCR of PAL1, PAL2, C4H, 4CL, HCT, CCoAOMT1, CCoAOMT2, F5H, COMT1, and COMT2 genes. The single peak observed presented the pure and single amplicon resulted from the assay. **Figure S4.** (A) Distribution of differentially expressed transcription factors (TFs); (B) Expression profiles of MYBs potentially related to lignin synthesis.

**Additional file 2: Table S1.** Overview of transcriptome sequencing and de novo assembly results. **Table S2.** Overview of transcriptome assembly showing length distribution of transcript and unigene. **Table S3.** Summary of functional annotation of contigs from BLAST searches against public databases. **Table S4.** KEGG enrichment analysis list. **Table S5.** Lignin related genes with RPKM values. **Table S6.** Differentially expressed transcription factors (TFs) list. **Table S7.** Primers used for qRT-PCR. **Table S8.** Primers used in the reference genes selection. **Table S9.** Average Ct, ΔC_T_, and coefficient of variation (CV) values used for the reference genes selection. The average values are shown for at least three replicates per gene and tissue type. Table S10. CDS sequences for monolignol biosynthetic genes.

## Data Availability

All data generated or analyzed during this study are included in this published article and the supplementary information files. The sequence data was deposited at NCBI database under the BioProject PRJNA550294.

## Abbreviations

*P. armeniacum*: *Paphiopedilum armeniacum*
Py-GC/MS: Pyrolysis-gas chromatography
DAP: Days after pollination
FTIR: Fourier transform infrared spectroscopy
DEG: Differentially expressed genes
NR: Non-redundant protein sequences
GO: Gene Ontology
COG: The Clusters of Orthologous Groups
KEGG: Kyoto Encyclopedia of Genes and Genomes
FDR: False Discovery Rate
PAL: Phenylalanine ammonia lyase
COMT: Caffeic acid O-methyltransferase
CCoAOMT: Caffeoyl-CoA 3-O-methyltransferase
TFs: Transcription factors
