# Supplementary material for: Transcriptome analysis provides insights into the non-methylated lignin synthesis in Paphiopedilum armeniacum seed
Source: BMC Genomics. 2020 Jul 29;21:524. doi: 10.1186/s12864-020-06931-1 (PMC7391499; doi:10.1186/s12864-020-06931-1)
Supplement: Supplementary file 1 — Additional file 1: Figure S1. (A) Correlation indices between different samples. (B) Principal component analysis performed on the 15 samples. A: 65 DAP, B: 87 DAP, C: 108 DAP, D: 122 DAP, E:150 DAP. Figure S2. Functional annotations of the unigenes of P. armeniacum seed transcriptome. (A) NR annotated species distribution map similar to the Paphiopedilum armeniacum transcriptome. Dendrobium catenatum shows the highest similarity. (B) GO function annotation. The most abundant functions are binding and catalytic activity in terms of molecular function and metabolic process and cellular process in terms of biological process. Figure S3. Additional data for qPCR assays. (A) The quality of RNA used for qPCR assessed by agarose gel electrophoresis. 1–4: 66 DAP, 5–8:87 DAP, 9–12: 108 DAP, 13–16: 122 DAP, 17–20: 150 DAP. Lanes 1 and 2 are the 28S and 18S RNA bands. (B) Quantification of RNA used for qPCR estimated by Nanodrop One and Agilent 2100 Bioanalyzer. (C) Melt curve from qPCR of PAL1, PAL2, C4H, 4CL, HCT, CCoAOMT1, CCoAOMT2, F5H, COMT1, and COMT2 genes. The single peak observed presented the pure and single amplicon resulted from the assay. Figure S4. (A) Distribution of differentially expressed transcription factors (TFs); (B) Expression profiles of MYBs potentially related to lignin synthesis. [file 12864_2020_6931_MOESM1_ESM.pdf]

Figure S1

A

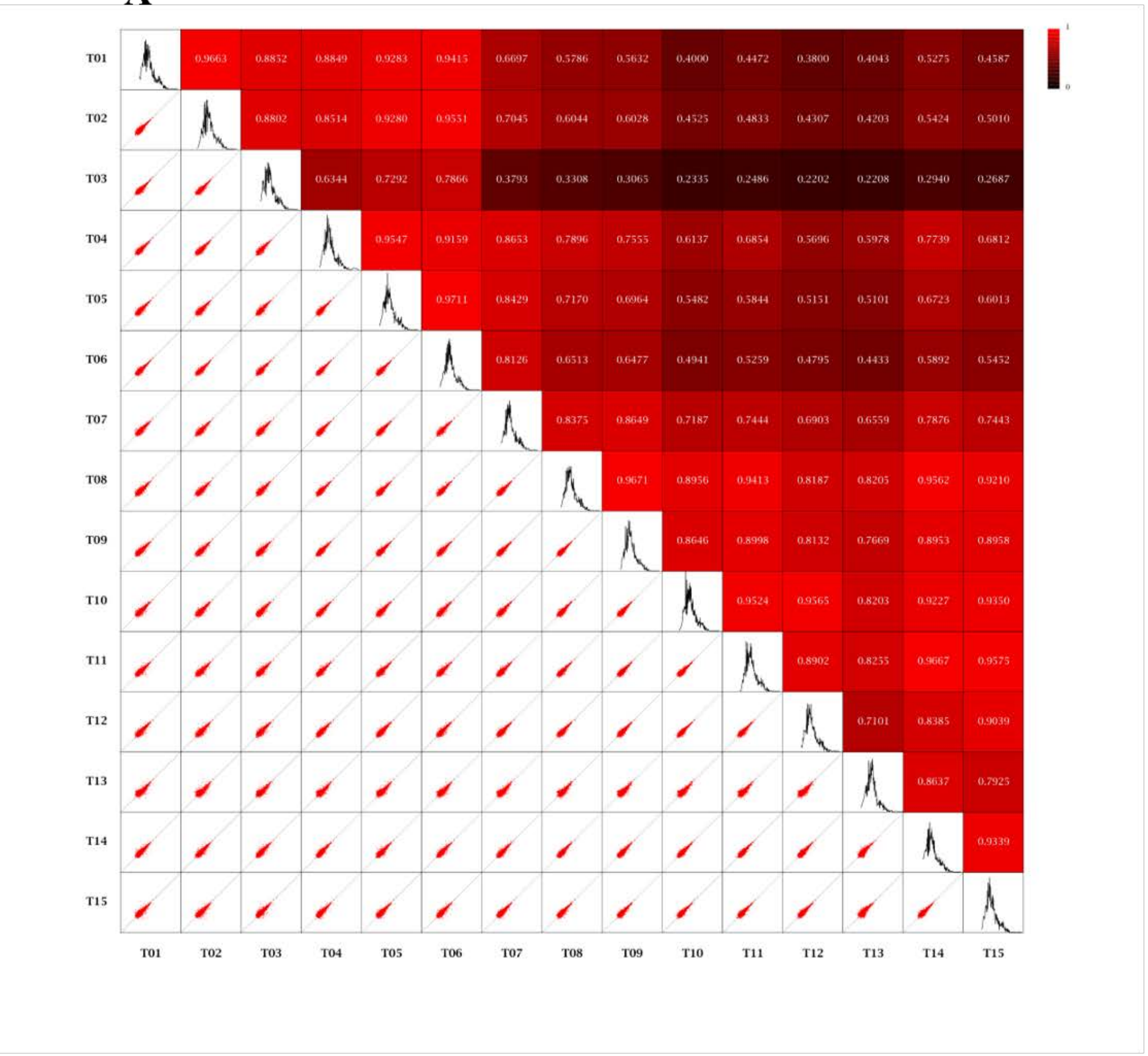
T01 T02 T03 T04 T05 T06 T07 T08 T09 T10 T11 T12 T13 T14 T15
T01 0.9663 0.8852 0.8849 0.9283 0.9415 0.6697 0.5786 0.5632 0.4000 0.4472 0.3800 0.4043 0.5275 0.4587
T02 0.8802 0.8514 0.9280 0.9551 0.7045 0.6044 0.6028 0.4525 0.4833 0.4307 0.4203 0.5424 0.5010
T03 0.6344 0.7292 0.7866 0.3793 0.3308 0.3065 0.2335 0.2486 0.2202 0.2208 0.2940 0.2687
T04 0.9547 0.9159 0.8653 0.7896 0.7555 0.6137 0.6854 0.5696 0.5978 0.7739 0.6812
T05 0.9711 0.8429 0.7170 0.6964 0.5482 0.5844 0.5151 0.5101 0.6723 0.6013
T06 0.8126 0.6513 0.6477 0.4941 0.5259 0.4795 0.4433 0.5892 0.5452
T07 0.8375 0.8649 0.7187 0.7444 0.6903 0.6559 0.7876 0.7443
T08 0.9671 0.8956 0.9413 0.8187 0.8205 0.9562 0.9210
T09 0.8646 0.8998 0.8132 0.7669 0.8953 0.8958
T10 0.9524 0.9565 0.8203 0.9227 0.9350
T11 0.8902 0.8255 0.9667 0.9575
T12 0.7101 0.8385 0.9039
T13 0.8637 0.7925
T14 0.9339
T15

B

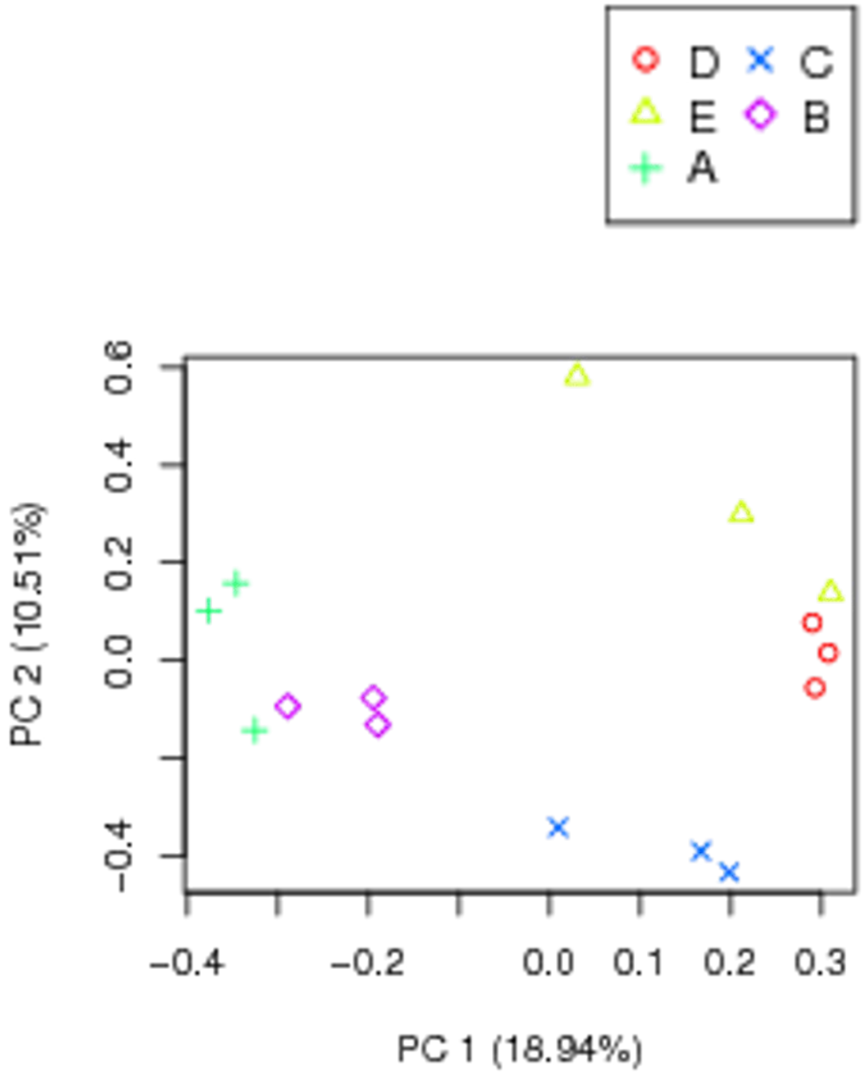
D
C
E
B
A
PC 2 (10.51%)
PC 1 (18.94%)
0.6
0.4
0.2
0.0
−0.4
−0.4
−0.2
0.0
0.1
0.2
0.3

Figure S2

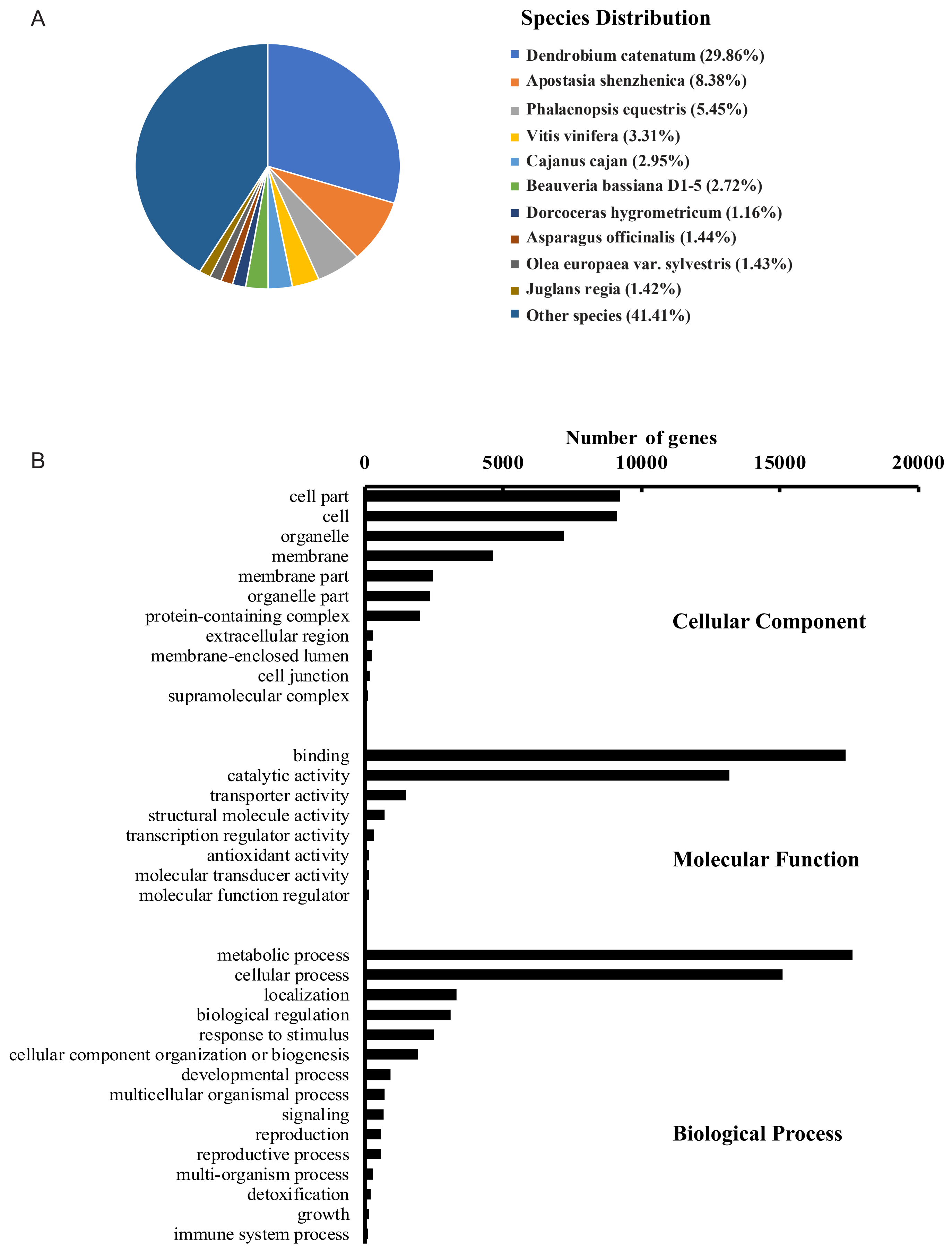
A
Species Distribution
Dendrobium catenatum (29.86%)
Apostasia shenzhenica (8.38%)
Phalaenopsis equestris (5.45%)
Vitis vinifera (3.31%)
Cajanus cajan (2.95%)
Beauveria bassiana D1-5 (2.72%)
Dorcoceras hygrometricum (1.16%)
Asparagus officinalis (1.44%)
Olea europaea var. sylvestris (1.43%)
Juglans regia (1.42%)
Other species (41.41%)
B
Number of genes
0
5000
10000
15000
20000
cell part
cell
organelle
membrane
membrane part
organelle part
protein-containing complex
extracellular region
membrane-enclosed lumen
cell junction
supramolecular complex
Cellular Component
binding
catalytic activity
transporter activity
structural molecule activity
transcription regulator activity
antioxidant activity
molecular transducer activity
molecular function regulator
Molecular Function
metabolic process
cellular process
localization
biological regulation
response to stimulus
cellular component organization or biogenesis
developmental process
multicellular organismal process
signaling
reproduction
reproductive process
multi-organism process
detoxification
growth
immune system process
Biological Process

Figure S3

A

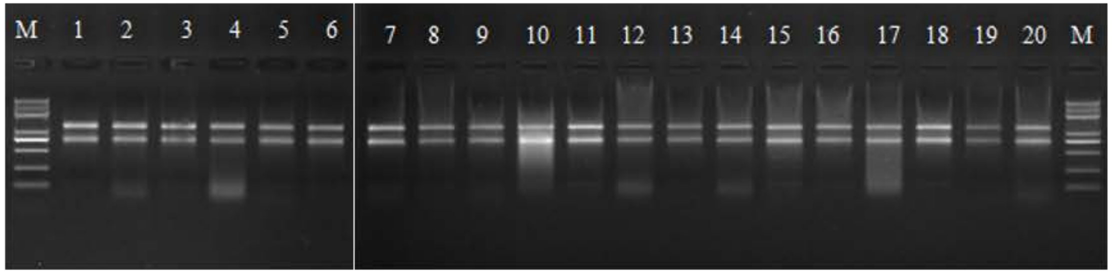

B

Sample	Concentration (ng/µl)	A_{260}/A_{280}	A_{260}/A_{230}	RIN
66DAP-1	218	2.18	2.14	8.4
66DAP-2	260	2.32	2.41	8.5
66DAP-3	186	2.21	3.21	8.5
66DAP-4	362	2.16	2.41	8.6
87DAP-1	234	2.60	2.92	7.9
87DAP-2	108	2.16	2.46	7.6
87DAP-3	154	2.49	2.75	7.7
87DAP-4	232	2.52	2.19	7.8
108DAP-1	216	2.20	2.35	7.7
108DAP-2	186	2.33	2.74	6.5
108DAP-3	170	2.66	2.50	7.7
108DAP-4	168	2.21	2.63	7.6
122DAP-1	114	2.38	2.48	7.4
122DAP-2	262	2.22	2.30	7.9
122DAP-3	194	2.31	2.26	7.0
122DAP-4	138	2.66	2.46	6.5
150DAP-3	172	2.10	2.32	6.9
150DAP-1	160	2.60	2.58	7.0
150DAP-2	70	3.50	3.50	7.3
150DAP-3	298	2.29	2.33	7.2

C

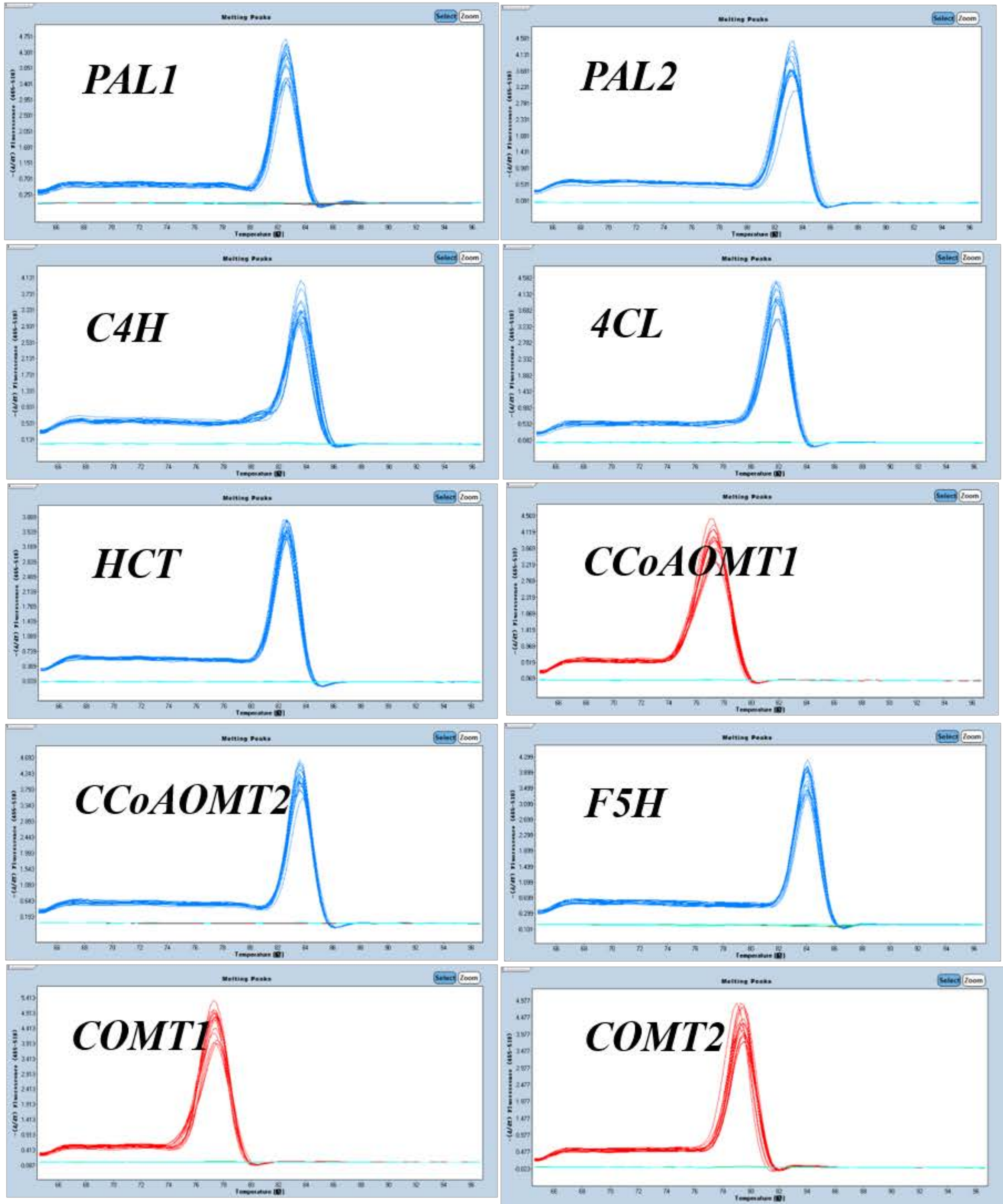

Figure S4

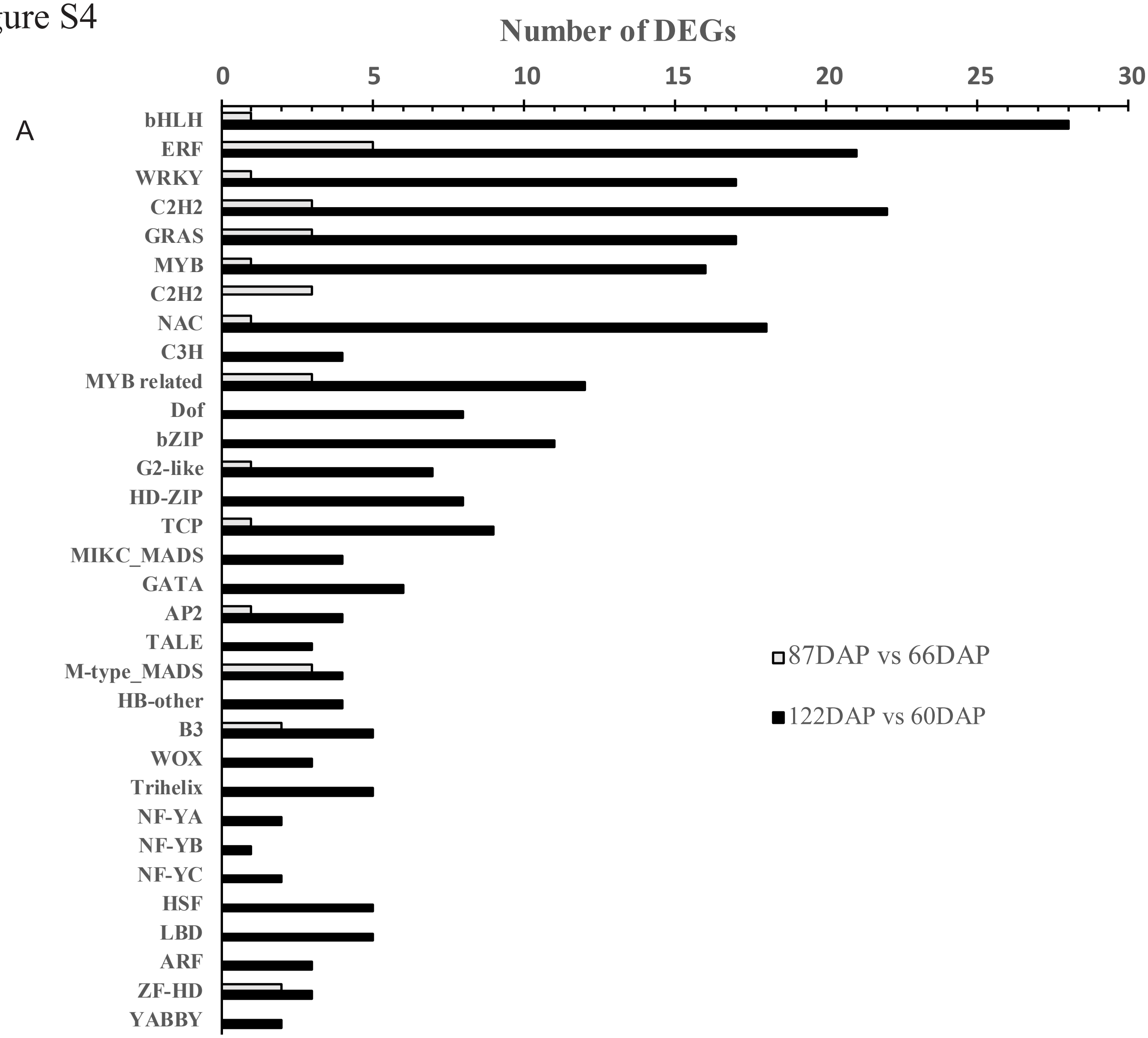

A
Number of DEGs
0
5
10
15
20
25
30
bHLH
ERF
WRKY
C2H2
GRAS
MYB
C2H2
NAC
C3H
MYB related
Dof
bZIP
G2-like
HD-ZIP
TCP
MIKC_MADS
GATA
AP2
TALE
M-type_MADS
HB-other
B3
WOX
Trihelix
NF-YA
NF-YB
NF-YC
HSF
LBD
ARF
ZF-HD
YABBY
87DAP vs 66DAP
122DAP vs 60DAP

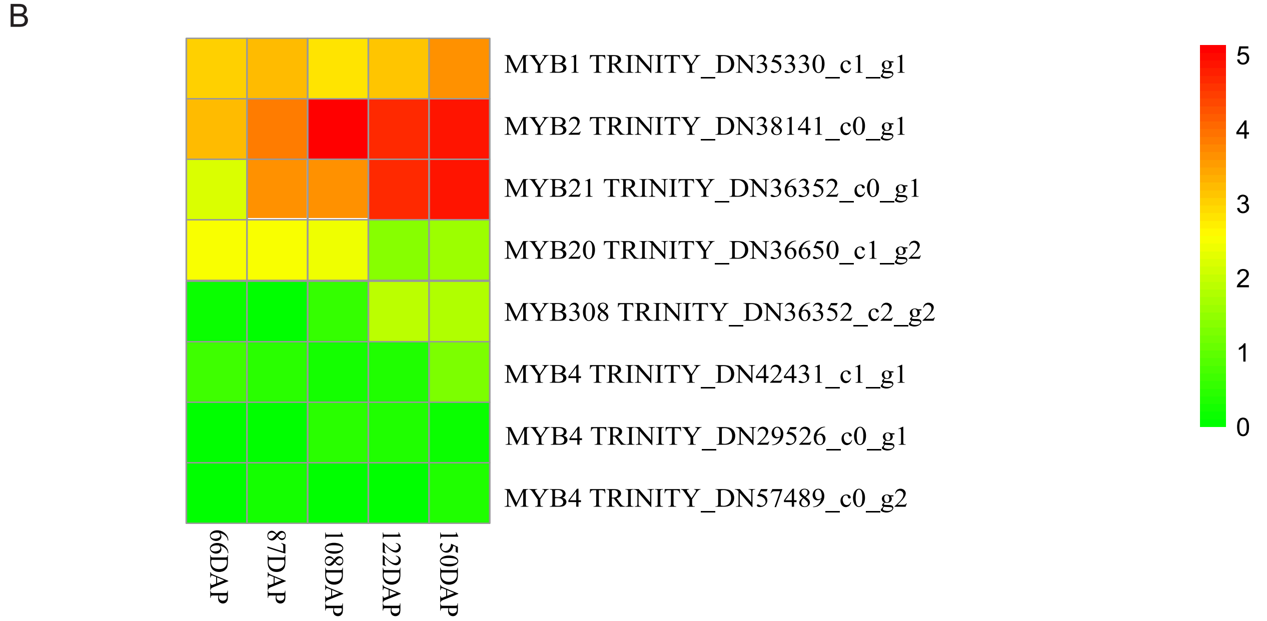

B
MYB1 TRINITY_DN35330_c1_g1
MYB2 TRINITY_DN38141_c0_g1
MYB21 TRINITY_DN36352_c0_g1
MYB20 TRINITY_DN36650_c1_g2
MYB308 TRINITY_DN36352_c2_g2
MYB4 TRINITY_DN42431_c1_g1
MYB4 TRINITY_DN29526_c0_g1
MYB4 TRINITY_DN57489_c0_g2
66DAP
87DAP
108DAP
122DAP
150DAP
5
4
3
2
1
0
